# Improving DTI Tractography by including Diagonal Tract Propagation

**DOI:** 10.1371/journal.pone.0043415

**Published:** 2012-09-06

**Authors:** Paul A. Taylor, Kuan-Hung Cho, Ching-Po Lin, Bharat B. Biswal

**Affiliations:** 1 Department of Radiology, UMDNJ-New Jersey Medical School, Newark, New Jersey, United States of America; 2 Institute of Neuroscience, National Yang-Ming University, Taipei, Taiwan; University Medical Center Groningen - UMCH, The Netherlands

## Abstract

Tractography algorithms have been developed to reconstruct likely WM pathways in the brain from diffusion tensor imaging (DTI) data. In this study, an elegant and simple means for improving existing tractography algorithms is proposed by allowing tracts to propagate through diagonal trajectories between voxels, instead of only rectilinearly to their facewise neighbors. A series of tests (using both real and simulated data sets) are utilized to show several benefits of this new approach. First, the inclusion of diagonal tract propagation decreases the dependence of an algorithm on the arbitrary orientation of coordinate axes and therefore reduces numerical errors associated with that bias (which are also demonstrated here). Moreover, both quantitatively and qualitatively, including diagonals decreases overall noise sensitivity of results and leads to significantly greater efficiency in scanning protocols; that is, the obtained tracts converge much more quickly (i.e., in a smaller amount of scanning time) to those of data sets with high SNR and spatial resolution. Importantly, the inclusion of diagonal propagation adds essentially no appreciable time of calculation or computational costs to standard methods. This study focuses on the widely-used streamline tracking method, FACT (fiber assessment by continuous tracking), and the modified method is termed “FACTID” (FACT including diagonals).

## Introduction

Diffusion tensor imaging (DTI) is a useful MR technique for non-invasively investigating structural properties of neural white matter (WM) by measuring the random and constantly occurring motion of fluid and aqueous tissue particles. Importantly, DTI tractography has been shown to provide a means for estimating the topology of WM tracts in vivo, increasing our understanding of brain physiology, pathology and structural connectivity in both clinical and research-oriented applications, e.g., [1]–[8]. For example, this technique has been shown to successfully reproduce many known WM pathways and to be useful in planning for neurosurgical operations. Tract-defined volumes also provide regions of interest (ROIs) for quantitative evaluation of axonal development and degeneration, such as with tract-based spatial statistics [9]–[12].

Several methods for performing DT tractography have been developed. Two main categories of techniques are streamline tracking (STT) [13]–[15] and tensor deflecting (TEND) [16]–[18]; the former variety directs tracts using information of only the first eigenvalue of **D**, and the latter utilizes that of all three eigenvalues. Common methods of propagating tracts include using either constant or variable step sizes (typically small compared to the voxel width) with Euler or higher order Runge-Kutta integration [13], [14], [19], or traversing between voxel boundaries in a single step, called FACT (fiber assessment by continuous tracking) [15], [20]. Tractography algorithms continue to be developed an refined, in order to deal with existing issues including: an inability to distinguish crossing and kissing fibers; difficulty following multiple pathways within a single voxel or multiple branchings; individual voxel errors due to signal noise or to numerical artifacts in reconstruction; uncertain termination conditions; a tendency of producing false negatives; and the accumulation of errors in propagating tracts. Additionally, non tensor-based models of diffusion have been developed, such as high-angular resolution (HARDI) techniques [21].

In this paper, we propose an elegant and simple means for improving existing tractography algorithms and for decreasing numerical artifacts by allowing tracts to propagate through diagonal trajectories between voxels instead of only rectilinearly to their facewise neighbors. The motivation of this change, which to the best of the authors' knowledge has not been previously implemented in any published tractography algorithms, is that the propagation of tracks should be independent (to the maximum degree possible) of the arbitrary orientation of scan geometry, which is represented by the coordinate axes. That is, the same tractographic output should be yielded by an object observed at various rotations under constant scanning conditions, or, equivalently, by an unmoved object scanned using various rotations of coordinates (ignoring minor variations due to random noise in the separate diffusion weighted measures).

In this study, we address numerical errors and inherent noise sensitivity which existing tractography algorithms exhibit due to their directional bias along scan coordinate axes. Namely, while the orientation of axonal fibers is entirely unrestricted in three-dimensional (3D) space (and, indeed, in a given whole-brain data set, a full range of directions is practically guaranteed to be represented), however numerical tracts are propagated only through facewise neighboring voxels along the orientations of the three coordinate axes. This restriction makes diagonally oriented or bending tracts difficult to follow and also biases the resulting structural connectivity. As discussed further below, this also makes algorithms more sensitive to local noise.

Through a series of tests using actual DTI scans (both human brains and a phantom) and an additional test using simulations of realistic data, we first show that “including diagonals” (ID) greatly decreases the dependence of the tracking on the arbitrary directions of the scan coordinate axes which exists in current algorithms. Moreover, the results from this study show that, both quantitatively and qualitatively, this adaptation reduces overall noise sensitivity and leads to significantly greater efficiency in scanning protocols; that is, the obtained results converge much more quickly (i.e., in a smaller amount of scanning time) to those of high SNR and spatial resolution data sets. Importantly, the inclusion of diagonal propagation adds essentially no appreciable time of calculation or computational costs to standard methods. In this study, we focus on the widely-used FACT method of tract propagation for streamline tracking (e.g., the citation count from Google for this paper [15] is >1500 citations total, with >200 citations since just 2011.) which is utilized, for example, in the widely used dtiStudio analysis package [22]. In this case, we term the improved method “FACTID” (FACT including diagonals).

## Materials and Methods

### 1 Ethics Statement

Participants from the university campuses in Taipei were enrolled after providing informed, written consent. The study was approved by the local Ethics Committee (Institutional Review Board of National Yang-Ming University), and it was conducted in accord with the Declaration of Helsinki.

### 2 Motivation and description of FACTID

**Figure 1:**
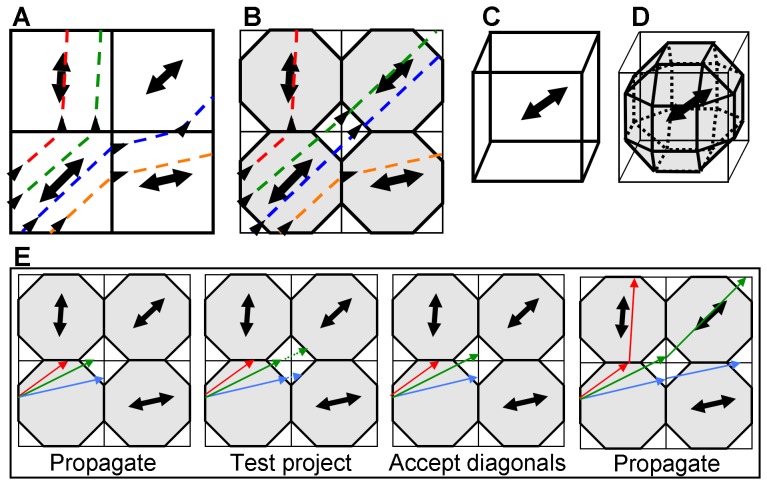
Schematic of tractography algorithms. Panel A shows an example of the propagation of four test tracks in 2D FACT algorithm (bold arrows show orientations of primary eigenvectors), where tracks cross from edge to edge of the lower left voxel and continue to facewise neighbors. Panel B shows the same system in the FACTID approach, where tracts propagate to the octagon surfaces in each voxel (bold line, gray area), allowing diagonal motion. The analogous 3D regions are shown for FACT and FACTID in panels C and D, respectively. Panel E illustrates the simple steps in the FACTID algorithm to allow for diagonal tract propagation (see text for details).

Fig. 1A shows a two dimensional (2D) example of tract propagation in FACT, with four test tracts (dotted lines, direction of propagation given by small arrowheads) entering and traversing the bottom-left voxel in the direction of greatest diffusion (shown as bold, dual-direction arrow). Each test tract becomes redirected at the voxel boundary, following the greatest diffusion in the next voxel (assuming the fractional anisotropy (FA) remains above a minimum threshold, and that the angular difference between the two voxels is below a given maximum). Of note in this example, is that there appears to be a tract running diagonally through the grid, which is largely missed by the tracking algorithm. If the coordinate axes were rotated 45 degrees, however, propagation could easily occur along this apparent tract. In other words, the orientation of the axes strongly affects the estimated tracts, leading to a high probability of numerical artifacts being present in the calculations. Furthermore, the propagating algorithm may be very sensitive to rotational perturbations to ellipsoids, which are a common consequence of signal noise: a small rotation of the bottom-left voxel may dramatically change the direction of propagation from horizontal to vertical, leading to a large path divergence. Thus, there are several cases where FACT is susceptible to producing numerical artifacts due to signal noise and to grid-dependence.

The solution proposed in this study, termed FACTID, is to allow tracts to propagate diagonally between voxels. This approach is shown for the same test tracts in Fig. 1B. In this scenario each tract propagates continuously to the octagon-shaped boundary in a given voxel (bold solid line) instead of the voxel boundary (thin solid line). Thus, the tracts from the bottom-left voxel will propagate vertically if they reach any of the three lower edges of the top-left voxel's octagon; horizontally if they reach any of the three leftward edges of the bottom-right voxel's octagon; and diagonally if they reach the single open edge of the upper-right voxel's octagon (though being redirected along the new eigenvector at the new voxel's edge in all cases). In this case it is possible to follow the diagonal tract, and results would remain similar even for a different orientation of coordinate axes. Also, small rotations of the main diffusion direction may produce less severe changes in the direction of the propagating path. The full 3D boundary-propagation surfaces for the old and new cases are shown for comparison in Fig. 1C and 1D, respectively; this method effectively increases the number of neighbors of a voxel from 6 in FACT (one for each face of the cube) to 26 in FACTID.

The simple additional steps in tract propagation are shown schematically for separate cases of a set of voxels in Fig. 1E (colors showing on different possible diffusion orientation of lower left square). In the first panel a given tract propagates until it reaches the current voxel edge. Then, a tract which reaches a corner region (i.e., the non-bold segment of the voxel edge) test projects to check which neighboring voxel is in line with its current trajectory (defined by intersecting the bold surface). If the tract intersects a diagonally-neighboring voxel, the tract extends over the short region to that voxel's edge. Finally, the tract may continue propagating per usual along the direction of the first eigenvector of the new voxel. Note that the simple additional steps to include diagonals do not appreciably increase either computing time or expense in switching from FACT to FACTID.

### 3 Evaluating and comparing algorithms

In several relevant scenarios, the FACT and FACTID algorithms would produce different results; however, the degree of variation would most likely depend on scanning conditions. Many of the differences between FACT and FACTID would be minimized by having extremely high SNR and spatial resolution. For example, in the former case there would be fewer instance of noise warping and rotating vectors; in the latter partial-volume effects would decrease, and the small horizontal and vertical steps of FACT would better follow diagonal tracts with less errors. In such “ideal” cases of both high SNR and spatial resolution, one would expect “reasonable” tractography algorithms to yield, for the most part, results which are predominantly convergent both with each other (that is, differences due to numerical approaches are minimal) and also with most of the actual pathways (small numbers of false positives and negatives). However, practical considerations of time and expense set finite limitations on scanning and therefore on both SNR and spatial resolution; the errors present at data acquisition will necessarily be accumulated and propagated in tractographic calculation, though in different manners and degrees in various algorithms. Previous studies have shown DTI tractography to be sensitive to both spatial resolution [23] and SNR/noise [24].

Therefore, in designing and testing an algorithm, one may view an important goal to be to produce results which converge as quickly as possible to the “ideal” cases of high SNR and spatial resolution. By minimizing numerical errors and artifacts, DTI studies can be made much more efficient by allowing best results with least scan time. In this study, we compare FACT and FACTID by analyzing their results as SNR and resolution are decreased from a level at which each algorithm produced mainly convergent results; relative noise sensitivity is also compared by adjusting the SNR of a data set with realistic noise and calculating the changes in tracts explicitly. Additionally, we show that results of FACTID are less affected by arbitrary coordinate axis orientation than FACT by testing both algorithms on a series of rotated scans (of constant SNR and spatial resolution).

### 4 Scanning protocols

Three healthy subjects (an adequate sample size for the purposes of algorithm testing and comparison in this study) gave informed consent and participated in this study consisting of three experiments. All MR images for human study were carried out on a 3 Tesla MR scanner (Trio, Siemens, Germany) equipped with a 32-channel phased-array heal coil. A twice refocused spin-echo echo planar imaging (EPI) sequence was used for DTI acquisition to minimize residual susceptibility effects [25]. Three b0 images and 30 diffusion weighted images with b = 1000 s/mm^2^ were acquired for each DTI data. The imaging parameters were: TR = 8800 ms, TE = 101 ms (99 ms for rotational invariance scans), FOV = 24×24 cm, BW = 1325 Hz/pixel and parallel acquisition (GRAPPA) with 2-fold acceleration.

Both FACT and FACTID tractography codes were implemented in custom C software. They were visualized using TrackVis [26], and additional analysis was performed using AFNI software [27], FSL [28] and Matlab (Mathworks, Natick, MA, USA). Data sets of the same spatial resolution were coregistered for motion correction; in all cases, motion was less than the length of one pixel. DTs were calculated from diffusion weighted images using nonlinear fits, implemented with AFNI. Whole brain, brute force tractography [24] was utilized, with tracts of interest and corresponding ROIs chosen by selecting tracts passing through specific seed ROIs. Within a voxel, seedpoints for commencing tracts were evenly distributed, the number typically dependent on spatial resolution in order to produce a constant number density per volume for comparison.

### 5 Methods of testing algorithms

In this study, we used five tests to examine several aspects of the convergence properties of the FACT and FACTID algorithms on human brain data sets. The following tests are used to: 1) compare the relative dependence of algorithms upon the orientations of coordinate axes; 2–3) compare the relative error propagation due to signal noise as SNR decreases; 4) compare the relative error propagation as spatial resolution decreases; and 5) compare results on known, underlying fiber bundles. Tests 1, 2, 4 and 5 use actual data sets (with last being a constructed phantom, described below), and the data sets in test 3 were created by including realistic noise of varied magnitude to real diffusion weighted (DW) data sets.

1. Invariance in rotation. Five DTI data sets with whole brain coverage and voxel size of 2.5^3^ mm^3^ were acquired with different slice orientation for the purpose of comparing the dependence of algorithms on the orientation of coordinate axes. The slices were rotated along (x, y, z) axes with angles of (0°, 0°, 0°), (0°, 0°, 10°), (0°, 0°, 20°), (0°, 0°, 40°), and (0°, 40°, 40°), respectively. Seed ROIs were mapped between data sets using linear registration (FMRIB's Linear Image Registration Tool, FLIRT) [29].
2. Convergence in SNR. Whole brain coverage of subject was obtained at constant 2 mm isotropic resolution. Scanning was repeated 16 times, allowing comparisons of various SNR by averaging acquisitions (after registration) of N_ave_ = 1, 2, 4, 8, 12 and 16. Changes in track number and location per voxel as a function of SNR (number of DW acquisitions averaged, N_ave_) were quantified.
3. Dependence on noise. The DTI ellipsoids of an actual data set were chosen to be a realistic “solution” brain image data set (here, the N_ave_ = 16, 2 mm data set from Test 2); noisy DWI copies of this data set were derived by adding Rician noise of various SNR_0_ ( = 10, 20 and 50; the subscript denotes that the quantity is measured with respect to the reference *b* = 0 signal) to synthesized anisotropic diffusion coefficient (ADC) measures along the same M = 30 gradient directions, similar to [24], [30], [31]. DTI ellipsoids were then calculated for each noisy data set, and tractography was performed (using 2^3^ seedpoints per voxel). Direct comparison of the alterations to number and locations of tracks per voxel were observed and quantified.
4. Convergence in spatial resolution. Whole brain coverage of same field of view (FOV) was obtained at isotropic resolutions of 3 mm, 2.5 mm and 2 mm, with N_ave_ 1, 3 and 11, respectively, for approximately constant SNR; the numbers of seedpoints per voxel for starting tracts were 6^3^, 5^3^ and 4^3^, respectively. Seed ROIs were mapped between data sets using FLIRT.
5. Phantom test. Publicly available DW data sets (30 gradients) from an existing phantom model were used (see details in [32], [33]). Having examined several options, here we selected a data set of highest resolution (3 mm isotropic) and DW factor which has been shown to be nearest optimal, b = 1500 s/mm^2^, for ellipsoidal modeling [34], [35]. One seedpoint per voxel was used, with a single tract kept through each of 16 specified ROIs. Quantitative metrics between an estimated tract and the “underlying truth” tract per ROI were calculated with a provided program: *dist* (L_2_-norm distance); *tan* (comparison of tangents); and *curv* (comparison of curvature) [33].

In all tests, standard FACT parameters were used. Tracts were propagated until reaching a voxel with FA<0.2 (except in the phantom test, which had no minimum FA) or until the turning angle between two the first eigenvectors of two successive voxels was θ>45°. Additionally, only tracts with path length >20 mm were retained for further analysis.

For comparison with existing and other tractography algorithms, the FACT-based dtiStudio [22] and DTI-Query [36] with Runge-Kutta integration were used for Test 1 and Tests 1–4, respectively. In DTI-Query, 4^th^ order Runge-Kutta (RK4) integration was used, with seeds placed at 2 mm intervals and tracts propagating with 1 mm steps (note that in all tests, voxel resolution was in a range of 2–3 mm).

For tests 1–4 (i.e., brain sets), images of the tracts which passed through specific ROIs were compared visually for comparison of fiber bundle location and extent. In tests 2–3 (those of perfectly aligned voxel boundaries; see the Discussion), three quantitative measures with slightly varied properties were calculated to quantify the similarity of tract volumes. The standard Dice coefficient, C_D_, was used to quantify the voxelwise overlap of tract-defined ROI maps [37]; the index is independent of the number of tracts passing through the same location and has a range of 0–1. For ROIs, A and B, the Dice coefficient is given as a fraction of sums over *n* total voxels:
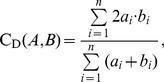
(1)where *a_i_* = 1 if *a_i_*∈A and *a_i_* = 0 otherwise (and similarly for *b_i_*). Following Dauguet et al., 2007 [38], seed regions which, by definition, are common to both ROIs were excluded from the calculation to avoid bias.

To include the relevant information of the number of tracts produced by each algorithm, we created a weighted version of the Dice coefficient, *C*_Dw_, as well as implementing the eta^2^ parameter [39]; both indices also have a range of 0–1. Since the number of tracts per voxel, *T_x,i_*, typically varied by orders of magnitude within a given data set, weights in the expressions utilized the logarithm of the number tracks, *w_x,i_* = log_2_(*T_x,i_*). The formulation of *C*_Dw_ is given by
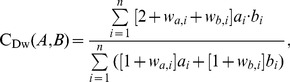
(2)and eta^2^ is given by
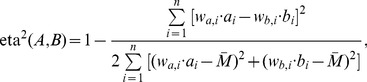
(3)where 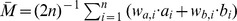. The difference in the two weighted similarity indices is that *C*_Dw_ compares the number of tracts in A and B independent of voxel location, while eta^2^ compares voxelwise number of tracts. That is, eta^2^ is unity only for perfectly overlapping regions if *T_a,i_* = *T_b,i_* for all *i*, while *C*_Dw_ is unity for perfectly overlapping regions if 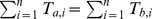.

The phantom test provides means for comparing FACTID with other, non-FACT-based algorithms, as well, through the scores of algorithms used in [33]. We note that these include both single- and multiple-DT and HARDI-based reconstructions; in this work, we are testing FACTID for single DT-based tractography only. However, it should be noted that FACT-based methods can be directly applied to HARDI and multiple-DT models, such as with MFACT in [40], and FACTID can be applied analogously. Single DT-based integration methods included streamline RK4 step-wise integration of a vector field first eigenvectors smoothed using trilinear interpolation. Additionally, Euler step-wise integration of tensor deflection propagation [16]–[18] (trilinear interpolation of tensor field) was implemented. A FACT-based method of propagation along principle eigenvectors was also implemented, using the version as described by [41], [42]. Other methods included multiple tensors, PAS-MRI [43] and HARDI models, as well as a global fitting mixture of Gaussian method [44], [45], though in this study we are investigating only single DT-based tractography approaches (with applications and extensions to other methods planned in future work, see Discussion).

## Results

**Figure 2:**
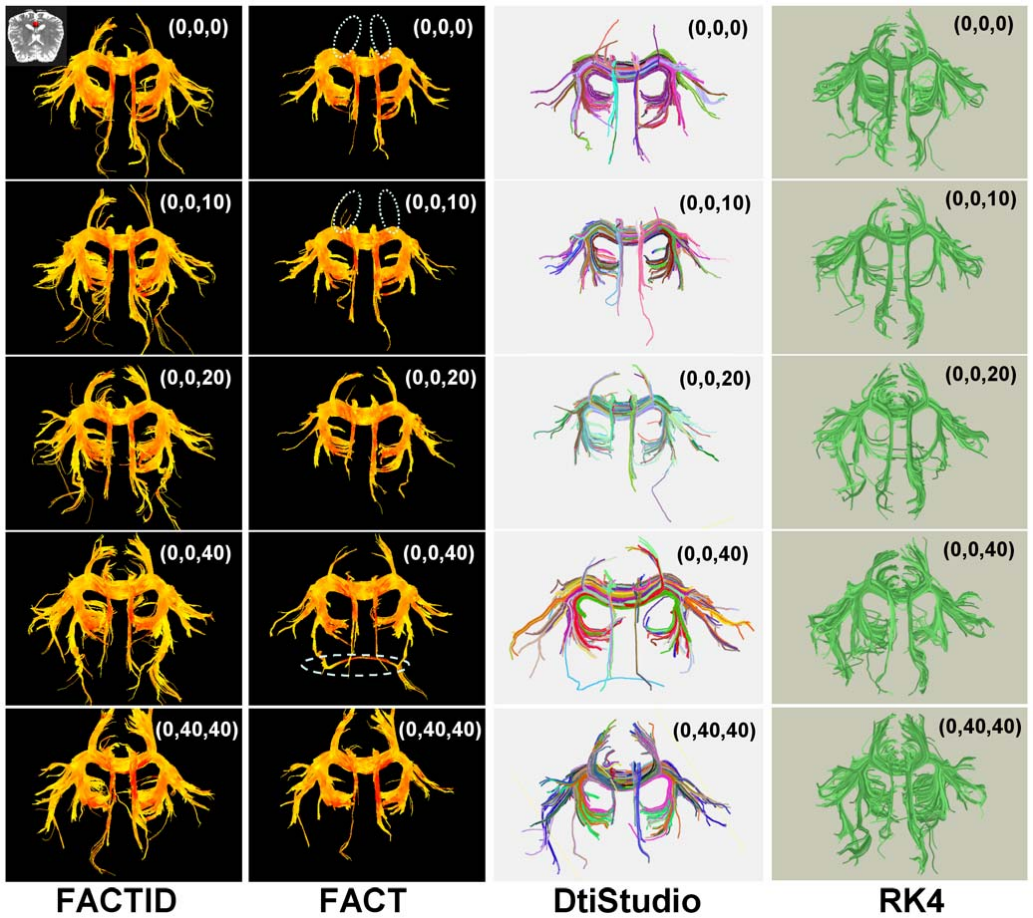
Algorithm comparison for rotation test. FACTID (col. 1) and FACT (col. 2) results are given for various rotations of coordinate axes. Corono-axial projections are shown of tracts intersecting a single ROI in the posterior corpus callosum which intersects the cingulum (shown in upper left). Dissimilarities in tract bundle location from average results are shown with dotted (missing) and dashed (additional) lines. Coloration by FA magnitude of each voxel, ranging from 0.2 (yellow) to 1.0 (red). Also shown for comparison are results for the tracts through the same ROI using dtiStudio-FACT (col. 3) and DTI-Query RK4 (col. 4), with distinct colorations from separate software. The former yields quite similar results to those of FACT in Column 2, and RK4 shows changes in fiber structure (though not with missing bundles).

In the first test (rotational invariance), data sets of constant resolution and SNR were obtained using slices which had been aligned with coordinate axes rotated by various angles. Fig. 2 shows a comparison of results for tracts passing through a single ROI, located in the posterior CC and intersecting the cingulum (ROI location shown in the figure). Visual comparison of tract bundle location and extent allows for an evaluation of the relative similarity amongst rotated sets for both FACT and FACTID. Locations where large tract bundles appear to be either missing or added in comparison to the group as a whole are highlighted in the figure. Quantitatively, the average values of similarity indices comparing the rotated sets (mapped to the non-rotated space using FSL) to the non-rotated are: *C*_D_ = 0.54, *C*_Dw_ = 0.67 and eta^2^ = 0.94 for FACT, and *C*_D_ = 0.54, *C*_Dw_ = 0.68 and eta^2^ = 0.95 for FACTID.

**Figure 3:**
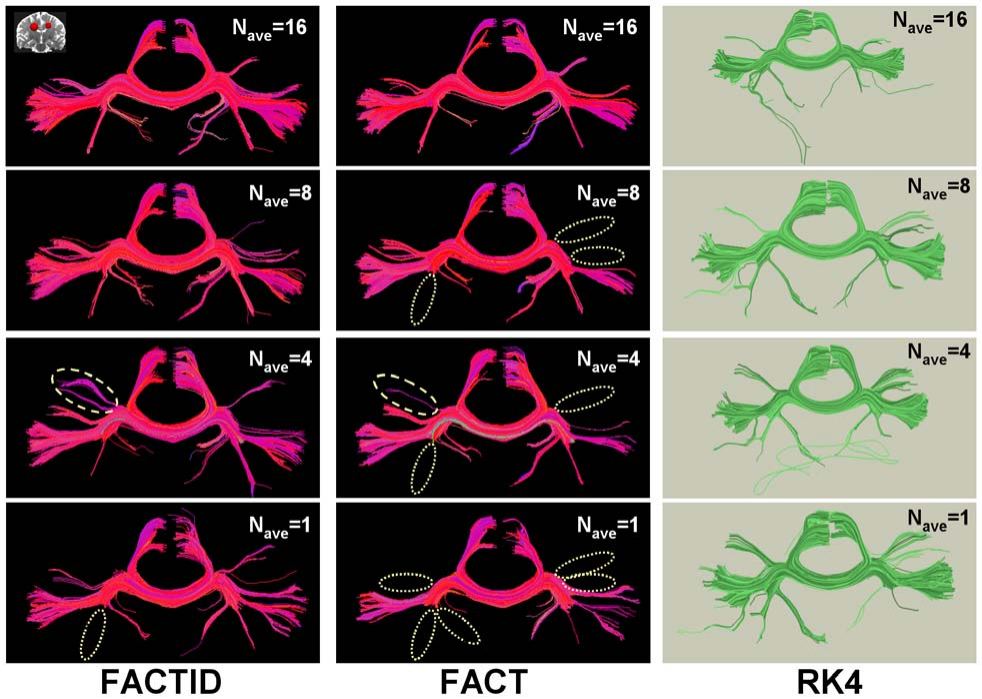
Algorithm comparison for various N_ave_. FACTID (col. 1) and FACT (col. 2) results are shown for various SNR (determined by N_ave_). Coronal projections are shown of tracts intersecting two mediofrontal ROIs (shown in upper left; AND logic). Dissimilarities in tract bundle location from the top panel are shown for N_ave_<16 results with dotted (missing) and dashed (additional) lines. Related Dice and eta^2^ coefficients are plotted in Fig. 4. Coloration by orientation of medial voxel of each fiber, with (x, y, z) associated with (red, green, blue). Also shown are RK4 results (distinct coloration), with fiber differences in inferior- and anterior-running tracts appearing.

**Figure 4:**
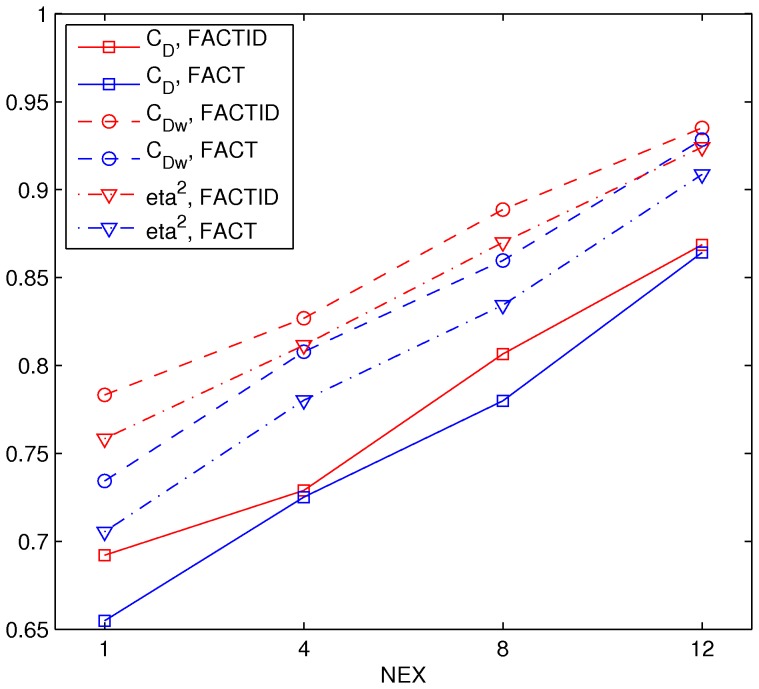
Similarity indices for various N_ave_. *C*_D_, *C*_Dw_ and eta^2^ (formulations given in text) of N_ave_<16 results compared with the highest SNR, N_ave_ = 16 case, for FACT and FACTID for tracts of interest (shown in **Fig. 3**).

In the second test (SNR convergence), tractography was performed on data sets of various N_ave_ (1 to 16) using both FACT and FACTID. Fig. 3 shows a comparison of results for tracts passing through two ROIs (AND logic) in the mediofrontal cortex and including the corpus callosum (CC) (locations shown in the figure). By visual inspection, at N_ave_ = 16 FACT and FACTID produce similar results in terms of extent and location of tract bundles, and similarity indices between the tracts of the two algorithms have high values, *C*_D_ = 0.86, *C*_Dw_ = 0.94 and eta^2^ = 0.93. Each column shows the effects of decreasing SNR (decreasing N_ave_) on the obtained tracts, with similarity indices between algorithms decreasing to *C*_D_ = 0.83, *C*_Dw_ = 0.92 and eta^2^ = 0.92 at N_ave_ = 1; locations of tract bundles which visual inspection show to be either missing or added in comparison with the highest SNR case are highlighted. Fig. 4 shows a comparison of relative similarity indices for FACT and FACTID results, plotting the values of the weighted and unweighted similarity indices for the tracts of the shown N_ave_(and also with N_ave_ = 12) with those of the N_ave_ = 16 case.

**Figure 5:**
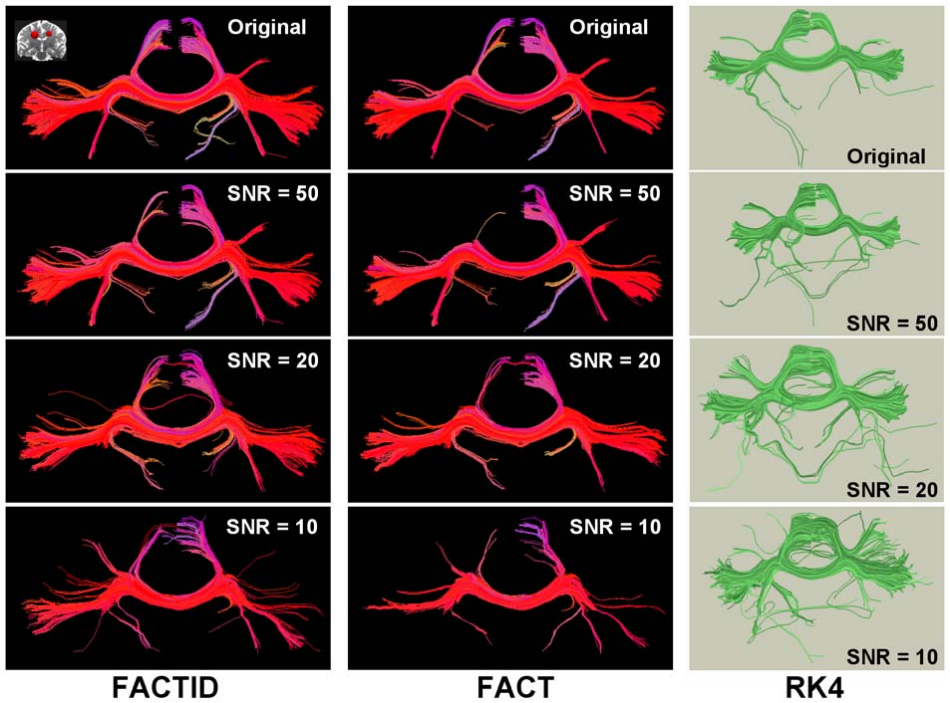
Comparison of algorithms for various SNR. FACTID (col. 1) and FACT (col. 2) results are shown (“noiseless” model at top, with realistic, noisy copies from DW simulations of decreasing SNR). Coronal projections are shown of tracts intersecting two mediofrontal ROIs (shown in upper left; AND logic). The number of increasing dissimilarities with decreasing SNR is apparent, with related Dice and eta^2^ coefficients plotting in Fig. 6. Coloration by orientation of medial voxel of each fiber, with (x, y, z) associated with (red, green, blue). Also shown are RK4 results (distinct coloration), with several changes in tract results apparent.

**Figure 6:**
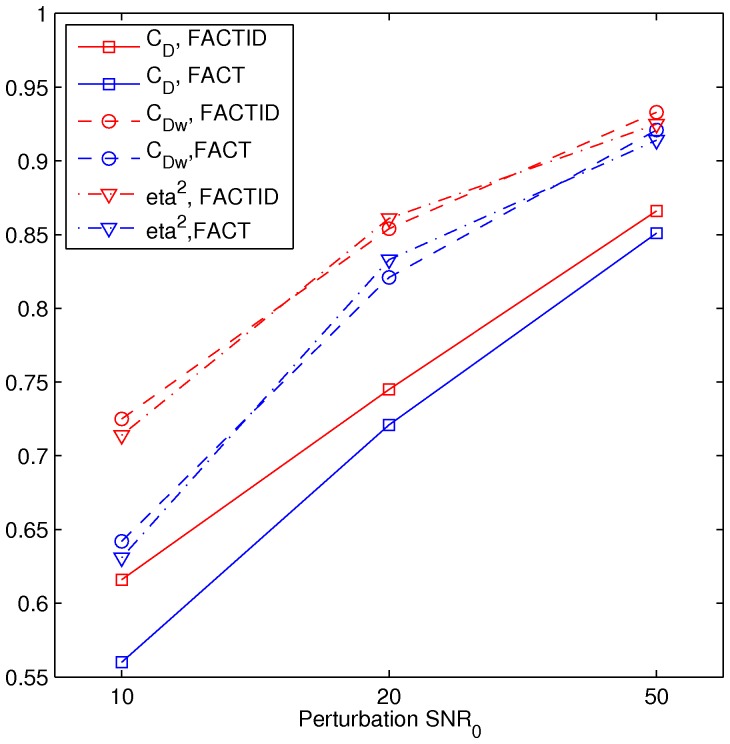
Similarity indices for changing SNR. *C*_D_, *C*_Dw_ and eta^2^ for FACT and FACTID are given for tracts of interest shown in Fig. 5 (comparison of noisy DW-derived simulations of decreasing SNR).

In the third test (noise dependence), the DT ellipsoids of a single scan were taken as a solution, and realistic sets of DWI data of SNR_0_ = 50, 20 and 10 were created. The results of tractography for the original data set and the noisy copies are shown in Fig. 5 using FACTID (left column) and FACT (right column); shown tracts pass through two ROIs (AND logic) in the mediofrontal cortex and including the CC (locations shown in the figure). Explicit differences in tract bundle locations are not highlighted in this case, due to the large number of differences at lowest SNR. The relation of tractographic results to SNR_0_ are shown quantitatively in Fig. 6 using the Dice and eta^2^ coefficients.

**Figure 7:**
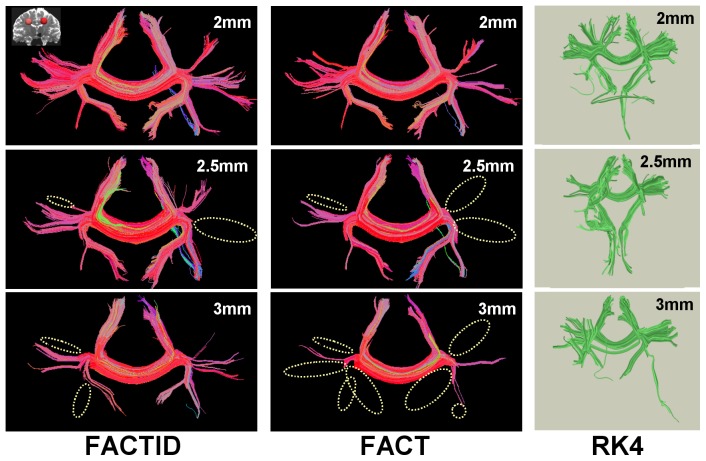
Comparison of FACTID (left column) and FACT (right column) algorithms for various spatial resolutions. Coronal projections are shown of tracts intersecting two mediofrontal ROIs (shown in upper left; AND logic). Dissimilarities in tract bundle location from the top panel are shown for results of 2.5 and 3 mm resolution with dotted (missing) lines. Coloration by orientation of medial voxel of each fiber, with (x, y, z) associated with (red, green, blue). Also shown are RK4 results (distinct coloration), with several differences in tract bundle results apparent.

In the fourth test (resolution convergence), tractography was performed on data sets of different spatial resolution, 2 mm to 3 mm (isotropic). A comparison of results are shown in Fig. 7 for tracts passing through two ROIs (AND logic) in the mediofrontal cortex and including the CC (locations shown in the figure). Visual comparison of tract bundle location and extent shows that similarity between FACT and FACTID results is greatest at high (2 mm) resolution and decreases as voxel size increases. Locations where tract bundles are missing at lower resolution in comparison to 2 mm resolution are highlighted in the figure, as well. Quantitatively, the similarity indices between the 2 mm- and 3 mm-resolution cases (mapped to the same space using FSL) are: *C*_D_ = 0.29, *C*_Dw_ = 0.43 and eta^2^ = 0.62 for FACT, and *C*_D_ = 0.38, *C*_Dw_ = 0.51 and eta^2^ = 0.66 for FACTID; between the 2 mm- and 2.5 mm-resolution cases, *C*_Dw_ = 0.82 and eta^2^ = 0.85 for both FACT and FACTID, with the Dice coefficients of *C*_D_ = 0.70 and 0.71, respectively.

**Figure 8:**
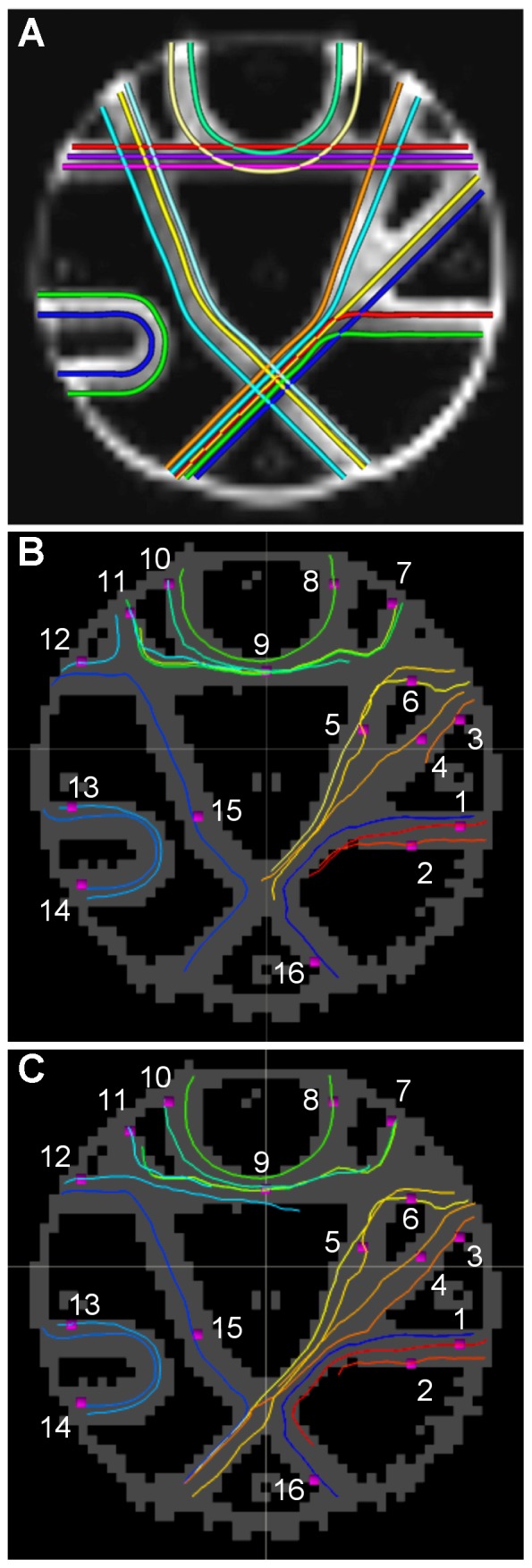
Comparisons of phantom test tractography results. 16 “underlying truth” tracts of the phantom are shown in (A), reproduced from Fig. 4C of Fillard et al. (2011) [32]. Tractography results from this study, represented by the longest tract passing through each test ROI (pink squares, numbered), are shown for FACT in (B) and for FACTID in (C). NB: identifying tract colors are the same in B and C, but different than in A. Quantitative scores per ROI are given in Table 1.

In the fifth test (known phantom), tractography was performed on a test phantom model, to produce known “underlying truth” tracts. A comparison of results are shown in Fig. 8, where panel A reproduces the underlying tracts (from Fig. 4 of [33]); panels B and C show FACT and FACTID results, respectively, having chosen the longest tract per ROI to represent the given tract (as generally multiple tracts were found passing through each ROI). Quantitative comparisons of tractography estimates and the underlying tracts are given (per ROI and overall mean) in Table 1. Measures of spatial deviation (*dist*), tangential variation (*tan*) and curvature differences (*curv*), are given for both the longest tracts and also for the best value per ROI, with lower values reflecting more similarity to the underlying tracts. Scores are generally similar, with FACTID producing lower mean scores overall in each category, and particularly much lower values for *dist* in ROIs 3 and 12. In both algorithms, some tracts are unable to propagate through or are redirected at crossing- and kissing-fiber junctions, though this occurred in many fewer cases for FACTID.

**Table 1:** Test metrics for the phantom results for the representative longest tracts per ROI (shown in Fig. 8), and also including the best values for each ROI (as generally each ROI contained more than one tract) in brackets.

	Phantom test metrics					
	dist [best dist]		tan [best tan]		curv [best curv]	
ROI	FACTID	FACT	FACTID	FACT	FACTID	FACT
1	18.1 [18.1]	23.4 [23.4]	45.3 [17.8]	25.7 [14.5]	0.16 [0.08]	0.31 [0.12]
2	29.2 [25.7]	24.8 [24.7]	32.7 [20.1]	20.7 [19.4]	0.22 [0.10]	0.18 [0.10]
3	5.1 [4.7]	53.4 [53.4]	17.8 [14.1]	20.9 [18.9]	0.16 [0.14]	0.23 [0.15]
4	18.5 [18.5]	15.3 [15.3]	13.2 [12.1]	13.9 [12.4]	0.12 [0.10]	0.16 [0.15]
5	7.8 [6.4]	15.2 [15.2]	37.5 [18.2]	45.2 [30.0]	0.18 [0.09]	0.19 [0.18]
6	45.2 [44.7]	45.5 [44.6]	61.7 [51.3]	60.4 [60.4]	0.28 [0.13]	0.23 [0.18]
7	54.5 [54.5]	54.4 [54.4]	67.6 [66.8]	65.5 [65.5]	0.20 [0.18]	0.20 [0.20]
8	5.0 [4.7]	4.8 [4.8]	15.2 [12.9]	15.2 [13.5]	0.12 [0.07]	0.12 [0.09]
9	11.4 [10.7]	10.4 [10.5]	43.5 [15.2]	43.3 [17.4]	0.16 [0.08]	0.25 [0.13]
10	9.2 [7.6]	10.6 [7.6]	32.5 [18.2]	46.9 [18.6]	0.14 [0.07]	0.20 [0.07]
11	56.3 [56.3]	54.9 [54.9]	53.7 [50.9]	56.2 [51.6]	0.23 [0.20]	0.28 [0.22]
12	18.9 [17.9]	56.4 [56.4]	18.9 [18.9]	76.0 [57.6]	0.19 [0.10]	0.17 [0.17]
13	3.8 [3.8]	3.1 [3.1]	15.9 [14.0]	14.6 [14.6]	0.09 [0.07]	0.11 [0.08]
14	4.5 [3.6]	5.1 [3.6]	21.0 [16.3]	23.1 [18.6]	0.11 [0.07]	0.13 [0.08]
15	16.9 [15.2]	15.4 [15.4]	58.1 [13.2]	53.7 [14.4]	0.18 [0.06]	0.22 [0.08]
16	60.2 [49.5]	60.3 [49.3]	65.6 [11.2]	65.6 [18.4]	0.32 [0.06]	0.36 [0.14]
Mean	22.8 [21.4]	28.3 [27.3]	37.5 [23.2]	40.4 [27.9]	0.18 [0.10]	0.21 [0.13]

The bottom row gives the mean value per column. NB: lower values per test reflect better values [32].

## Discussion

Tractography approaches continue to be developed and refined, in part due to the fact that there is currently no “gold standard” test for algorithms, though test phantoms, such as utilized here, provide useful data sets for comparison with known underlying construction. Even histological studies cannot provide definitive comparison, though they do provide important verification of results as well as specify limitations. In this study, an improvement to standard tractography methodology, FACT, was proposed and tested, that of allowing diagonal propagation of tracts through voxels. The motivation of the new approach, FACTID, was to decrease the dependence of algorithms on the direction of arbitrary coordinate axes, thereby decreasing numerical errors in results. Three criteria were examined for studying the results of two tractography algorithms on real data sets and on one realistic set. Both FACT and FACTID were compared in terms of important properties: their relative invariance to rotation of axes (i.e., relative coordinate independence), their rate of convergence with increasing SNR, and their rate of convergence with increasing spatial resolution.

Both visual inspection and quantitative comparison (with the weighted and unweighted Dice coefficients and eta^2^, as well as test phantom metrics) were employed in the analysis. We note that across the cases of changing spatial resolution and particularly the angles of coordinate axes, the use of *C*_D_, *C*_Dw_ and eta^2^ also becomes convolved with the mapping of the tenuous structures. However, for all tests visual comparison of fiber location provided meaningful evaluation of relevant similarities and differences, with the excision or addition of tract bundles with changing protocols being an important form of contrast and dissimilarity between algorithms.

In the rotation invariance test, the degree of homogeneity of results was significantly greater using the FACTID algorithm. Of course, variation in the outputs of either algorithm is expected due to interscan variability and potentially to changes of partial voluming in corresponding voxels as axes were rotated; this can be noted in some of the change in coloration (FA value) across the scans at analogous tract location (Fig. 2). Variation is seen in the density of some of the FACTID tracts as well as in the location of some of the lateral extensions of the CC. Qualitatively, the FACTID results appear to have more “split ends” than FACT, most likely due to the accumulation of gradual changes allowed by diagonal propagation as opposed to the more discrete changes of the latter. In the FACT cases, significant alterations to tract structure are observed, where the superior- and posterior-directed extensions of the CC were not reproduced by in two orientations (while they were using FACTID), and the lengths of the cingulum bundles varied considerably.

Both tests of convergence produced comparable outcomes: as the quality of scanning conditions was decreased (in SNR or spatial resolution), larger differences appeared in the FACT tractography results than in those of FACTID. In Tests 2, 3 and 4, the number of missing FACT tract bundles quickly increased (Figs. 3, 5 and 7). Quantitatively, in the SNR and noise tests the FACTID indices of similarity to the highest N_ave_data were consistently higher, as well (Figs. 4 and 6). While there were also tract bundles that were not reproduced by FACTID in cases of decreased SNR and resolution, in general the results of the algorithm appeared to retain most of the features of the highest quality scans (i.e., having fewer number of fiber bundle errors and consistently greater similarity indices).

The relative constancy of tractography results using FACTID in Test 1 suggests minimal dependence or near independence of the algorithm on coordinate axis orientation, which had been a motivation for its inception. This is critical in tractography, as tracts in the brain follow many trajectories at all possible angles to the scan axes. Moreover, the relative consistency of results when varying SNR or spatial resolution suggest a reduced accumulation of error due to signal noise or numerical effects. FACTID may also be able to follow faster-bending curves than FACT for the same maximum turning angle, in cases where the eigenvector of the facewise-neighbor is at too large an angle to the propagating path, while the eigenvector of the diagonal voxel remains in the “cone” of allowed values. The numerical costs of moving from an implementation of FACT to FACTID are negligible, as well.

In the limits of high SNR, where noise-induced rotations are small, and of high spatial resolution, in which small horizontal and vertical steps approximate a smooth curve well, FACT and FACTID should yield nearly identical results (in convergent cases, approximated as part of Tests 2, 3 and 4). However, as resolution decreases, similar to increasing the step size, Δx, when applying the Euler method to solve differential equations, errors will accumulate more quickly, particularly when WM pathways are not aligned with the coordinate axes. Decreasing SNR accentuates these errors for all reconstruction algorithms. Here, we have demonstrated that these errors occur with a reduced degree and with less bias on coordinate direction when including diagonals, converging more quickly to high SNR, high spatial resolution results. A similar implementation of including diagonal propagation may improve other tractography approaches as well, such as those utilizing TEND or Runge-Kutta integration.

The phantom tests provide a useful comparison of the algorithms against predetermined results. Generally, FACTID performed as approximately as well or better than FACT for all 16 test tracts, particularly in the important *dist* metric (Table 1), which quantifies deviation of the estimated path itself from the underlying fibers. In several cases, FACTID produced significantly better scores, in large part due to its successful propagation through crossing- and kissing-fiber regions, which FACT was typically unable to accomplish (Fig. 8). Though, the FACTID algorithm did produce some misdirected and truncated tracts, as well.

The phantom test has been used on several tractography algorithms (see op. cit. within [33] for details), some with the DT model and some with HARDI, such as orientation distribution functions [21], which often require a large number of scans (only sets of 30 DWIs at multiple *b*-values and resolutions were available, however, for this phantom data set). In general, these methods provided the best scores across the phantom tests. For many fibers, FACTID produced results comparable with these values, though improvement can be made in some cases for navigating complex fiber regions. For most ROIs, FACT results were similar or better than most DT-models (such as streamline propagation with Runge-Kutta integration [13], [46] or TEND propagation with Euler integration [16], [46]) in terms of *dist* values, such as tract-ROIs 3 and 12, and tying top scores for with various others having various performance (e.g., ROIs 5, 8 and 14); it should be noted that FACTID had a much lower than top score than other methods on ROI 6, which contained several crossing and one kissing fiber. The general improvement of scores with FACTID are likely due to decreased error accumulation from lower noise sensitivity and reduced directional bias (as suggested by Tests 1–4). Of course, future work will be done to further improve the algorithm, particularly in navigating crossing/kissing fibers (such as required in ROI 6). It would be interesting as well to perform this phantom test in an analysis of false positives in order to test the variability of connectivity which various algorithms produce.

While the FACTID algorithm appears to decrease the speed of error accumulation and to decrease some numerical features due to axis orientation, of course artifacts still remain in the results. Cases of potential “false positive” tracts appeared in the N_ave_ = 4 data set of the SNR test (Fig. 3); though, a pathway appeared in similar location in the FACT results of the same data, as well. The FACTID algorithm is still susceptible to many of the same imperfections of FACT, such as difficulty in distinguishing crossing and kissing fibers (though, as seen in the phantom test, including diagonals reduced these errors) or to propagate through regions of low FA (where a real tract may continue, but a crossing fiber disorients the diffusion anisotropy). Future improvements to FACTID may include additional rules for dealing with such difficulties or more sophisticated stopping criteria.

From the results of the study, it is apparent that the inclusion of diagonals in FACTID makes significant improvement to the facewise-propagating FACT algorithm, and we recommend the former's inclusion in standard and widely-used software. Improvements to results in terms of decreased noise sensitivity and reduced propagation bias would apply to any FACT-based algorithm. Additionally, we are currently working on implementing FACTID with both probabilistic tractography and higher-resolution diffusion methods, such as HARDI, DSI, etc. The inclusion of diagonals is likely to be useful in reducing numerical errors in other tractography algorithms, as well, particularly those due to coordinate-directional bias. It must be noted that several tractography algorithms propagate with steps smaller than voxel-traversing distances (such as Euler and RK4 integration methods), and diagonal propagation may occur by chance depending on the specific step location, size and direction. However, including diagonals would provide systematic propagation in a greater number of directions and for any step size, which has been shown to be a significant improvement in this study. We plan to investigate this in future studies of other algorithms with various step sizes, expecting improvements on various scales at essentially no extra computational cost.

## Conclusions

Therefore, while work remains to be done to continue improving FACT-based tractography, the simple inclusion of diagonal propagation has been shown to have a number of advantages which make FACTID a much preferred algorithm. Quantitative and qualitative investigation have shown that FACTID produces results which are more efficient with regards to SNR and to spatial resolution (as limited by scanning time) and more independent of arbitrary coordinate directions (and therefore, more consistent across the brain and scanning sessions). Also, including diagonal propagation typically decreased the sensitivity of results to MR noise. Therefore, in all tractographic applications, FACTID decreases requisite scanning time for studies and simultaneously increases reliability and robustness of results.
